# Surgical debridement for acute invasive fungal rhinosinusitis during the pre-engraftment phase of allogeneic hematopoietic stem cell transplantation: Two case reports

**DOI:** 10.1097/MD.0000000000012696

**Published:** 2018-10-19

**Authors:** Hao-yu Cheng, Lei Yuan, Jing-bo Wang

**Affiliations:** a Department of Haematology, China Aerospace Central Hospital; b Department of Haematology, Peking University Third Hospital, Beijing, 100191, China.

**Keywords:** acute rhinosinusitis, allogeneic hematopoietic stem cell transplantation, fungal, pre-engraftment, surgical debridement

## Abstract

**Rationale:**

Surgical intervention may be not a contraindication for acute invasive fungal rhinosinusitis (AIFR) during the pre-engraftment period of allogeneic hematopoietic stem cell transplantation (allo-HSCT).

**Patient concerns:**

We present 2 cases involving patients with AIFR in the pre-engraftment phase of allo-HSCT.

**Diagnoses:**

Both patients received surgical debridement combined with systemic antifungal treatment. The biopsies identified the diagnosis of AIFR in these 2 cases.

**Outcomes:**

The 2 patients obtained normal hematopoiesis without recurrence of AIFR.

**Lesson:**

Our experience with these 2 cases suggests that prompt endoscopic surgical debridement is not an absolute contraindication for allo-HSCT recipients with AIFR during the pre-engraftment period. If permitted, urgent, radical, and aggressive but careful endoscopic debridement should be performed together with systemic antifungal treatment once AIFR has been diagnosed or suspected.

## 1 Introduction

Acute invasive fungal rhinosinusitis (AIFR), which is characterized by a pathogenic fungal invasion of the nasal cavity and paranasal sinuses, is highly fatal, with overall mortality rates approaching 50% to 80% among immunocompromised patients.^[1,2]^ Notably, the prognosis of nonhematopoietic stem cell transplantation (HSCT) recipients with AIFR improved significantly with a combination of surgery and powerful fungal active antibiotic therapy, and the strong recommendation of surgical intervention for AIFR in the most recent Infectious Diseases Society of America guideline is supported by moderate-quality evidence.^[3]^ However, data regarding AIFR during the pre-engraftment phase of allo-HSCT are rather limited. Accordingly, experience with AIFR management under such circumstances is deficient. Furthermore, although surgical intervention for AIFR is well accepted, it is extremely technically challenging. Herein, we report 2 cases of AIFR treated with endoscopic surgical debridement in conjunction with antifungal agents.

## 2 Case reports

### 2.1 Case 1

**Figure 1:**
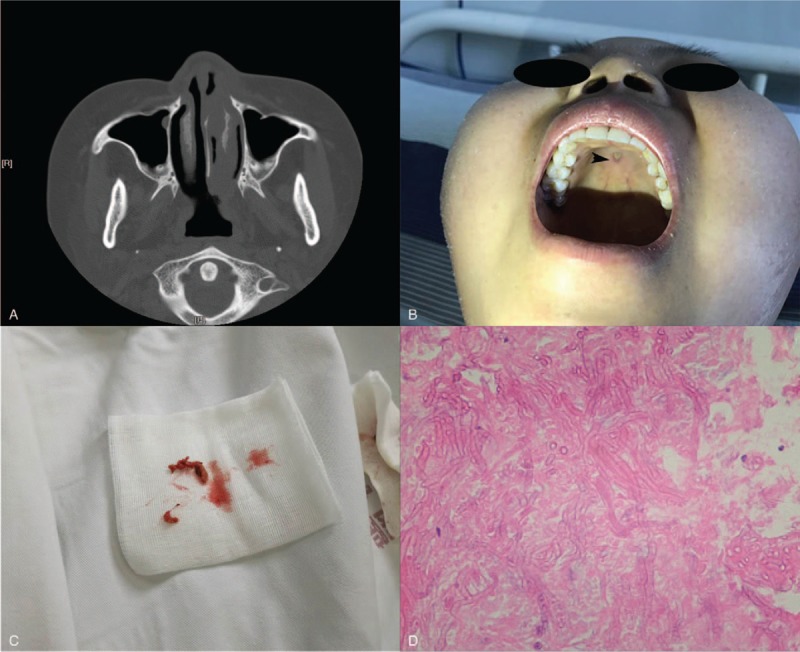
A, A computed tomography scan of the paranasal sinuses showed mucosal thickening in her maxillary sinus and a leukemic mass in her nasal cavity. B, An erosion on the hard palate (arrow). C, Necrotic tissue excised. D, Presence of *Talaromyces marneffei* in the tissue (HE stain ×400).

A 20-year-old woman with *FLT3/ITD* mutation-positive relapsed/refractory acute myeloid leukemia (AML) was transferred to our institute in June 2017. Following a diagnosis of AML-M5, she received 4 cycles of primary chemotherapy and 1 cycle of unsuccessful salvage chemotherapy for recurrent disease and complex pneumonia, and voriconazole to cure a cutaneous ulceration. After admission, she developed acute appendicitis and recovered following a laparoscopic appendectomy. A computed tomography scan of the paranasal sinuses showed mucosal thickening in her maxillary sinus and a leukemic mass in her nasal cavity (Fig. 1A). Despite a blast ratio of >90% in her marrow and pathologically proven extramedullary disease in both her central nervous system (CNS) and nasal cavity, we performed salvage allo-HSCT with donor tissue from her father. This study was approved by the institutional Ethics Committees of China Aerospace Center Hospital and conducted in accordance with the ethical guidelines of the Declaration of Helsinki. Written informed consent was obtained from the patient for the publication of this case report and accompanying images.

For graft-versus-host disease (GVHD) prophylaxis, a total body irradiation–based myeloablative conditioning regimen comprising antithymocyte globulin (ATG), cyclosporine, mycophenolate mofetil, and a short methotrexate course was used. The patient developed febrile neutropenia, headache, nasofacial pain and swelling on day +3, and a white blood cell count of 240/mm^3^ and platelet count of 36,000/mm^3^ indicated the need for transfusion. A physical examination showed ulceration and grayish tissue necrosis in the anterior nasal cavity, with an erosion on the hard palate (Fig. 1B arrow). However, the leukemic mass in the nasal cavity had decreased significantly since conditioning.

Because we strongly suspected AIFR, we administered liposomal amphotericin B (L-Amp B, AmBisome, 3 mg/kg i.v. daily), imipenem (1.0 g i.v. q8 hours), and a donor-derived neutrophil transfusion. As no symptomatic improvement occurred during the first 24 hours, an experienced otolaryngologist performed an urgent aggressive endoscopic sinonasal debridement. Subsequently, a low-temperature plasma radiofrequency ablation and radical necrotic tissue excision were performed after the patient's platelet count exceeded 30,000/mm^3^ via transfusion (Fig. 1C). Pathology indicated the presence of *Talaromyces marneffei* in the tissue, concordant with the blood culture result (Fig. 1D). Although the species is known to respond well to triazole,^[4]^ L-Amp B therapy, which yields superior CNS penetration, was maintained to avoid an adverse interaction between high-dose triazole and calcineurin inhibitor therapy. After a 10-day therapeutic regimen, the maintenance therapy switched to oral voriconazole as the patient's symptoms significantly improved. Neutrophil and platelet engraftment occurred on days +11 and +14, respectively. Although the leukemia unfortunately deteriorated, no recurrence of fungal infection was observed until treatment was terminated at 4 months post-HSCT.

### 2.2 Case 2

**Figure 2:**
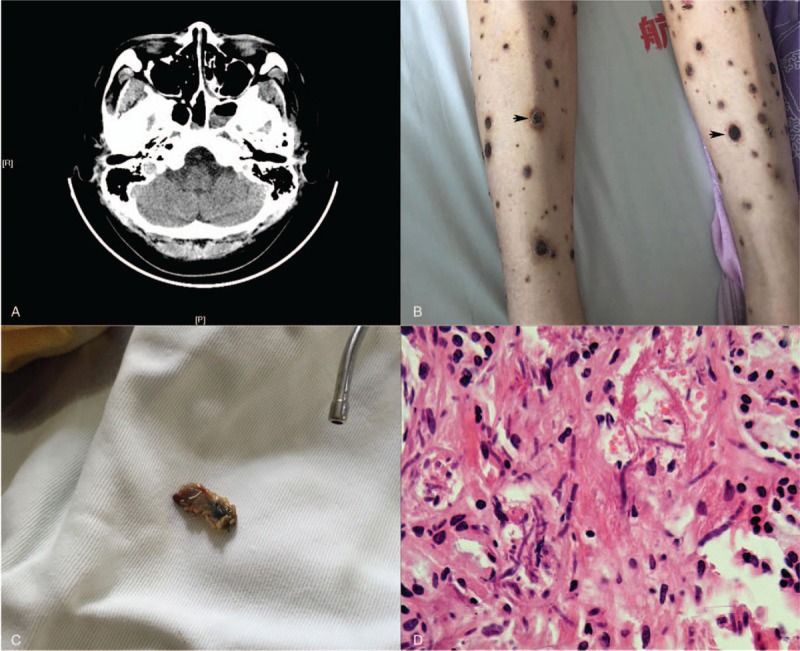
A, A magnetic resonance imaging of the paranasal sinus showed no abnormalities. B, Mucosal ulceration and purplish skin nodules on the bilateral legs (arrow). C, Necrotic tissue excised. D, Presence of *Fusarium* species in the tissue (HE stain ×400).

A 21-year-old woman with refractory relapsed *FLT-3/TKD* mutation–positive AML was admitted to our institute in April 2015. Although she had achieved a complete remission (CR) after the initial induction chemotherapy, this was lost after 4 additional cycles of medium-dose cytarabine consolidation therapy. Despite 2 unsuccessful cycles of standard reinduction chemotherapy, leukemic CNS involvement was controlled via intrathecal therapy. Postadmission, low-dose cytarabine-based cytoreduction chemotherapy was used to treat the rapidly progressing disease. Although magnetic resonance imaging of the paranasal sinus showed no abnormalities (Fig. 2A), the patient exhibited tumor lysis syndrome, disseminated intravascular coagulation, diffuse alveolar hemorrhage, and transient heart failure, which were controlled after 2 weeks of therapy. Despite a 1-year history of laparoscopic resection for a left-sided cystic kidney, normal renal function had been maintained since the onset of AML.

While receiving intravenous voriconazole treatment after cytoreduction chemotherapy, she developed fever, vision difficulty, nasofacial pain, and nasal congestion, as well as mucosal ulceration and purplish skin nodules with progressive necrosis on her bilateral legs (Fig. 2B arrow). We switched the patient from voriconazole treatment to L-Amp B (3 mg/kg i.v. daily) and conducted an urgent frontal sinusotomy. The biopsy specimen culture was positive for *Fusarium* species. This study was approved by the Institutional Ethics Committees of China Aerospace Center Hospital and conducted in accordance with the ethical guidelines of the Declaration of Helsinki. Written informed consent was obtained from the patient for the publication of this case report and accompanying images.

Despite a marrow blast ratio >90%, salvage allo-HSCT with busulfan-based myeloablative conditioning comprising ATG, cyclosporine, mycophenolate mofetil, and methotrexate for GVHD prophylaxis was performed. Despite secondary prophylaxis with continuous L-Amp B, she experienced febrile neutropenia and headache with blackish tissue necrosis in her frontal nasal cavity on day-2. Two consecutive blood and 1 pharyngeal culture identified the presence of *Fusarium* species.

Once multiple transfusions allowed the patient's platelet number to meet the requirement for surgery, the otolaryngologist performed another aggressive surgical debridement with low-temperature plasma radiofrequency ablation and excised the necrotic tissue (Fig. 2C). The patient's symptoms improved 2 days later. Pathology confirmed the diagnosis of fusarial sinusitis (Fig. 2D). L-Amp B maintenance therapy was not replaced with voriconazole until day +60. Neutrophil and platelet engraftment occurred on days 8 and 12, respectively. Despite the extended use of L-Amp B and unilateral nephrectomy, the patient maintained normal renal function and remained symptom-free and had sustained a CR after a 39-month follow-up at the time of this submission.

## 3 Discussion

To our knowledge, few reports describe surgical debridement in the context of AIFR management during the allo-HSCT pre-engraftment period. Surgical interventions during this a period are technically challenging, given the patients’ poor condition, immunocompromised state, and thrombocytopenia. However, our experience suggests the feasibility and necessity of surgical interventions for allo-HSCT recipients even during the pregrafting phase. In a study of 37 cases of surgical debridement in patients with various hematological diseases or diabetes,^[5]^ Sercan et al. maintained thrombocyte counts of >50 000/mm^3^ and hemoglobin (Hb) levels >10 g/dL before initiating surgery. Eliashar et al^[6]^ established a surgical platform for AIFR that emphasized the importance of careful and thorough preoperative preparation, including appropriate platelet counts and Hb levels, as well as an overnight stay in the intensive care unit, hypotension general anesthesia by an experienced anesthesiologist, in terms of surgical success. In our study, a senior otolaryngologist, local anesthesia, and a platelet count >30,000/mm^3^ via transfusion were sufficient to guarantee the surgery. Although good preparation requires more time, this would extend AIFR and increase infection-related mortality, and a lengthy intensive care unit stay would remarkably increase the risk of additional opportunistic infection during the pre-engraftment period. Nevertheless, we successfully and safely performed endoscopic surgical debridement in the 2 above-described patients. We therefore suggest the importance of urgent surgery under the guidance of an experienced otolaryngologist for patients with AIFR who have achieved a sufficient platelet count.

The extent of the infection or severity of the underlying disease may narrow the window in which surgery can be performed for AIFR. Piromchai and Thanaviratananich^[7]^ reported that the interval before treatment initiation was the most statistically significant predictor of mortality. Consistent with a previous report by Sercan of a strong association between hard palate involvement, a late-stage disease manifestation, and mortality,^[5]^ the patient in our first case developed a hard palatal erosion synchronously with ulceration and grayish tissue necrosis in the anterior nasal cavity after febrile neutropenia. Accordingly, we advise prompt radical surgical debridement for recipients who have been diagnosed with or are highly suspected to have AIFR.

Radical aggressive debridement must be performed to maximally reduce the probability of disease recurrence. First, the traditional surgical strategy encourages aggressive surgical resection to the bleeding margin, which requires skull base resection and cerebral debridement.^[8–10]^ Consistent with this, our second case underwent a second operation for disease recurrence after an initial nonradical frontal sinusotomy. This suggests that a bloody surgical margin is essential for HSCT recipients with AIFR during the pre-engraftment phase. However, skull base resection and cerebral debridement present challenges, because these procedures would postpone the engraftment; furthermore, the large wound would increase HSCT-related mortality. Previously, Mutchnick et al^[11]^ reported the successful management of a pediatric AIFR case with orbital and intracranial involvement without orbital exenteration or cerebral debridement. Consistent with that report, we did not resect the involved palate in the first case, and AIFR did not recur until the leukemia deteriorated and treatment was terminated 4 months after HSCT. Therefore, we suggest that endoscopic radical debridement, when combined with systemic antifungal therapy, could sufficiently control the disease.

There were limitations to our study. Because of technical limitations, the antifungal treatment was based on the clinicians’ experience owing to the fact that antifungal susceptibility tests on the resected tissues were not performed following the surgery. In addition, our study would benefit from having the cooperation of patients and experienced otolaryngologists. Addressing these limitations in future studies would shed further light on the real impact of surgical debridement on the management of AIFR during the pre-engraftment phase of allo-HSCT.

Our experience with these 2 cases suggests that prompt endoscopic surgical debridement is not an absolute contraindication for allo-HSCT recipients with AIFR during the pre-engraftment period. If permitted, urgent, radical, and aggressive but careful endoscopic debridement should be performed together with systemic antifungal treatment once AIFR has been diagnosed or suspected.

## Author contributions

**Data curation:** Hao-yu Cheng.

**Writing – original draft:** Lei Yuan.

**Writing – review & editing:** Jing-bo Wang.
